# Can clinicians identify community-acquired pneumonia on ultralow-dose CT? A diagnostic accuracy study

**DOI:** 10.1186/s13049-024-01242-w

**Published:** 2024-08-07

**Authors:** Anne Heltborg, Christian Backer Mogensen, Helene Skjøt-Arkil, Matthias Giebner, Ayham Al-Masri, Usha Bc Khatry, Sangam Khatry, Ina Isabell Kathleen Heinemeier, Jonas Jannick Andreasen, Sanne Sarmila Sivalingam Hariesh, Tenna Termansen, Anna Natalie Kolnes, Morten Hjarnø Lorentzen, Christian Borbjerg Laursen, Stefan Posth, Michael Brun Andersen, Bo Mussmann, Camilla Stræde Spile, Ole Graumann

**Affiliations:** 1 Department of Emergency Medicine, University Hospital of Southern Denmark, Kresten Philipsens Vej 15, 6200 Aabenraa, Denmark; 2 Department of Regional Health Research, University of Southern Denmark, Odense, Denmark; 3 The Learning and Research Centre, University Hospital of Southern Denmark, Aabenraa, Denmark; 4 Department of Internal Medicine, University Hospital of Southern Denmark, Sønderborg, Denmark; 5 Department of Internal Medicine, University Hospital of Southern Denmark, Kolding, Denmark; 6 Department of Respiratory Diseases, Odense University Hospital, Odense, Denmark; 7 Department of Clinical Medicine, University of Southern Denmark, Odense, Denmark; 8 Department of Emergency Medicine, Odense University Hospital, Odense, Denmark; 9 Department of Radiology, Herlev and Gentofte Hospital, Herlev, Denmark; 10 Department of Radiology, Odense University Hospital, Odense, Denmark; 11 Department of Radiology, Aarhus University Hospital, Aarhus, Denmark; 12 Department of Clinical Medicine, Aarhus University, Aarhus, Denmark

**Keywords:** Community-acquired pneumonia, Community-acquired infections, Ultralow-dose CT, Diagnostic imaging, Antimicrobial stewardship, Emergency medicine

## Abstract

**Background:**

Without increasing radiation exposure, ultralow-dose computed tomography (CT) of the chest provides improved diagnostic accuracy of radiological pneumonia diagnosis compared to a chest radiograph. Yet, radiologist resources to rapidly report the chest CTs are limited. This study aimed to assess the diagnostic accuracy of emergency clinicians’ assessments of chest ultralow-dose CTs for community-acquired pneumonia using a radiologist’s assessments as reference standard.

**Methods:**

This was a cross-sectional diagnostic accuracy study. Ten emergency department clinicians (five junior clinicians, five consultants) assessed chest ultralow-dose CTs from acutely hospitalised patients suspected of having community-acquired pneumonia. Before assessments, the clinicians attended a focused training course on assessing ultralow-dose CTs for pneumonia. The reference standard was the assessment by an experienced emergency department radiologist. Primary outcome was the presence or absence of pulmonary opacities consistent with community-acquired pneumonia. Sensitivity, specificity, and predictive values were calculated using generalised estimating equations.

**Results:**

All clinicians assessed 128 ultralow-dose CTs. The prevalence of findings consistent with community-acquired pneumonia was 56%. Seventy-eight percent of the clinicians’ CT assessments matched the reference assessment. Diagnostic accuracy estimates were: sensitivity = 83% (95%CI: 77–88), specificity = 70% (95%CI: 59–81), positive predictive value = 80% (95%CI: 74–84), negative predictive value = 78% (95%CI: 73–82).

**Conclusion:**

This study found that clinicians could assess chest ultralow-dose CTs for community-acquired pneumonia with high diagnostic accuracy. A higher level of clinical experience was not associated with better diagnostic accuracy.

**Supplementary Information:**

The online version contains supplementary material available at 10.1186/s13049-024-01242-w.

## Background

Community-acquired pneumonia (CAP) is a common cause of hospitalisation worldwide [1], and antibiotic treatment is often indicated to treat and prevent potential deterioration to sepsis or respiratory failure. Even more often, CAP is a differential diagnosis requiring diagnostic investigations in acutely hospitalised adults [2, 3]. Fast and accurate CAP diagnosis is relevant for optimal patient treatment; hence, a core element to support in-hospital antimicrobial stewardship [4].

Diagnostic imaging is required in CAP diagnosis in a hospital setting, as clinical signs and symptoms alone have insufficient diagnostic ability [5]. Despite lack of accuracy, chest radiographs are most commonly used [1, 6]. Chest computed tomography (CT) is diagnostically superior to chest radiographs [6, 7]. Yet, standard-dose CT is not suitable for routine CAP diagnosis due to the cancer risk related to higher radiation exposure [8].

Reduced-dose chest CT is an emerging imaging modality for lung tissue that minimises risks and ethical concerns by lowering radiation exposure. Depending on the dose reduction, the investigation is referred to as low-dose or ultralow-dose CT (ULD-CT), with no strict definition of effective radiation doses [9]. In recent studies, the accuracy in detecting various pulmonic pathologies with ULD-CT compared to standard-dose CT has been good, including the identification of changes consistent with pneumonia [10–12]. These studies applied mean effective radiation doses between 0.05 and 0.26 mSv. This is similar to the radiation exposure from a regular chest radiograph (approximately 0.1 mSv [13]. For comparison, standard dose CT of the chest provides mean radiation exposures around 5–7 mSv [13, 14].

The addition of a reduced-dose chest CT reported by a radiologist has been shown to increase diagnostic certainty of clinicians treating patients suspected of having CAP in hospitals [7, 15]. However, radiologist resources are limited, especially in the emergency department (ED). Thus, adding the ULD-CT for patients suspected of CAP includes a potential delay in radiological reporting. Hypothetically, that could delay patient treatment or impair the clinician’s decision-making basis when starting treatment. If the ED clinicians were able to perform the first ULD-CT assessment for CAP changes, it would increase early diagnostic certainty and remove some time pressure from the ED radiologists.

The primary aim of this study was to investigate the diagnostic accuracy of ED clinicians’ independent assessments of the presence of CAP on chest ULD-CTs from acutely admitted patients with a clinical appearance suggesting CAP using an ED radiologist’s assessments as reference. Secondary aims was to investigate the association between level of clinical experience and diagnostic accuracy, reliability of the clinicians’ assessments, and their confidence in their assessments.

## Methods

### Study design

The study was a cross-sectional diagnostic accuracy study using retrospectively collected data. It was part of the umbrella project: Infectious Diseases in Emergency Departments (INDEED), on improving acute infection diagnostics to support antimicrobial stewardship in hospitals [16].

The study was registered by the Danish Data Protection Agency (no. 20/60508). Ethical approval was obtained from the local ethics committee, and all patients provided written and oral informed consent. Reporting was guided by the Standards for Reporting of Diagnostic Accuracy Studies (STARD) guidelines [17].

### Setting

The study utilised ULD-CTs conducted on patients recruited from a Danish ED setting. Here, acutely hospitalised non-trauma patients were referred to a medical specialty (surgery, cardiology, neurology, or acute medicine) prior to medical assessment. The study focused on staff and patients referred to the acute medicine unit, where patients suspected of CAP are primarily assessed. Following the initial clinical assessment, further diagnostic investigations are ordered based on the tentative diagnoses, including referral to diagnostic imaging. A treatment plan is aimed at being set within the first four hours of the hospital stay. Patient inclusion was conducted by study assistants on weekdays between 8 a.m. and 8 p.m. from March 2021 to February 2022. Resources for patient inclusion were not available during night-time hours.

In this study, the ULD-CT data was utilised outside of the clinical environment after finalised patient inclusion. The planning, preparation, and collection of clinician assessments were conducted in the second half of 2022. The clinicians contributed to the study in their non-working hours.

### Study population

The study population was a subsample of the INDEED project CAP population [16]. Patients were eligible if the receiving clinician suspected CAP at the initial clinical assessment, with no further diagnostic tests available. No specific requirements for symptoms or findings were set. Patients over 40 years old were eligible for a ULD-CT investigation to avoid unnecessary radiation exposure to younger adults. The most important exclusion criteria were: a) patients with verified SARS-CoV-2 infection within two weeks (to avoid a pandemic-related dominance of this disease in the study population); b) recent hospitalisation within 14 days (to avoid hospital-acquired infections); c) patients currently undergoing immunosuppressive or antineoplastic treatments (as they represent a population in need of specialist evaluations, and rarely candidates for restrictive antibiotic treatment). Further details on participation criteria is available in the protocol [16].

For the current study, we extracted consecutively included patients with an ULD-CT available from one inclusion site (Hospital Lillebaelt, Denmark) for consistency in ULD-CT images. No emphasis was put on image quality or the presence of CAP, as the study population should reflect a realistic flow of patients suspected of CAP.

### Test methods

The index test was ten clinicians’ individual ULD-CT assessments for CAP. All clinicians were affiliated with the acute medicine unit of an ED. They represented two different levels of experience: five junior doctors with 0–1 year of clinical experience and five consultants in emergency medicine and/or internal medicine (not including pulmonologists, to avoid bias from their experience with assessing CT).

Prior to study assessments, all clinicians attended a five-hour web-based, interactive course focusing on assessing ULD-CT for typical pneumonic opacities. Other acute findings (pneumothorax, pleural effusion, and pulmonary oedema) were briefly covered as well. The course was organised and conducted by a professor in radiology (OG), with considerable experience in both teaching and research and clinical work with ULD-CT. The course included a short theoretical presentation, cases for individual assessment and plenary discussion, an individual test with ten ULD-CT case assessments, and a follow-up with feedback on test cases.

A web-based picture archiving and communication system (PACS) by Collective Minds Radiology (Sweden) was used for anonymised ULD-CT presentation.

The clinicians’ assessments were registered on a template in Research Electronic Data Capture (REDCap). The primary content was a binary assessment of the presence of pneumonic opacities consistent with CAP, and confidence in this assessment stated on a 7-point Likert scale. In addition, presence of pneumothorax, pleural effusion, and pulmonary oedema were to be registered (template available in Additional file 1).

The reference standard was a yes/no assessment of ULD-CTs for presence of CAP by one ED radiologist with 10 years of experience (CSS). Findings interpreted as pneumonia were consolidations that were not in a tumour or nodular pattern, tree-in-bud patterns, poorly defined peri-bronchial nodules observed in bronchopneumonia, and ground-glass opacifications. The reference assessment was part of a more thorough ULD-CT assessment. Thus, the radiologist’s assessment template in REDCap was not identical to the clinicians’ template (relevant parts of the template are available in Additional file 2). The radiologist also performed an assessment of image quality of the entire chest CT scan. However, the image quality of the lungs was always sufficient to address common point-of-care questions such as pneumothorax and pneumonia.

All assessors, including the radiologist, were aware that ULD-CTs were conducted to investigate for suspected CAP. They were blinded to other clinical data, comorbidities, previous and follow-up imaging, and ULD-CT assessments by the other assessors.

### Analysis

#### Summary statistics were used to describe the data

Assisted by a statistician, sensitivity, specificity, and predictive values of the clinicians’ CAP assessments were calculated using generalised estimating equations (GEE) with a logit link function to account for correlations in assessments within each rater. The same model was applied for accuracy calculations on subgroups defined by image quality, chronic pulmonary disease diagnoses, and clinicians’ confidence in their assessments. We used a z-test to examine the statistical difference in diagnostic performance related to the two levels of clinical experience.

Rater reliability was calculated as both kappa (Cohens kappa for pairwise comparison, Conger’s kappa for multiple raters fully crossed design [18]) and percent agreement. Kappa interpretation was: κ ≤ 0: no agreement, 0.01–0.20: none to slight, 0.21–0.40: fair, 0.41–0.60: moderate, 0.61–0.80: substantial, and 0.81–1.00: almost perfect [19].

STATA statistical software (BE17.0, STATA Corporation, Texas) was used for analyses.

### Sample size

The sample size was based on the precision of the sensitivity and specificity estimate, which was set at 15% point as we hypothesised sensitivity and specificity to be 85%, and wanted the lower limit of the confidence interval to be at least 70%. As the confidence intervals was on bootstrap we employed Monte Carlo simulation. From this, we needed 128 patients.

The number of clinicians was determined based on rational considerations, aiming to obtain reasonable face validity and heterogeneity among clinicians with respect for the clinician time required for the study.

The number of duplicate case assessments for intrarater reliability calculations was calculated to be 12 (expected kappa 0.85 and at least 0.5). Thereby, all clinicians ended up making 140 ULD-CT assessments. The clinicians were not informed of the presence of duplicates. Duplicates were presented with at least 90 other ULD-CTs in between and not in sequence. Assessments from one of each duplicate was randomly discarded prior to other analyses.

### Ultralow-dose CT specifications

A GE Revolution CT scanner (GE Healthcare, Waukesha, US) was used for the non-enhanced ULD-CTs. The applied ULD-CT protocol administered a mean effective dose of 0.27 mSv to a test sample using an identical scanner. Standard parameters of the ULD-CT protocol are presented in Table 1. Detailed information on technical specifications was published in a technical note by Mussmann et al. [20].

**Table 1:** Standard parameters of the chest ULD-CT protocol

Parameter	ULD-CT
Tube voltage	100 kV
Tube current modulation range	10–740 mA
Noise Index	85
CTDI_vol_	0.41
Scan time	0.5 s
Pitch	0.5
Collimation	128 × 0.625 mm
Kernel	Lung, mediastinum
ASIR-V	50%

ULD-CT: Ultralow-dose computed tomography

CTDIvol: Volumetric Computed Tomography Dose Index

ASIR-V: Adaptive Statistical Iterative Reconstruction

As a part of the umbrella project (INDEED), the ULD-CT protocol was validated for CAP diagnosis against a standard-dose chest CT conducted in the same sequence. Additionally, we collected data on chest radiographs which most patients underwent as part of standard care. The readings for the pneumonia from ULD-CT and standard-dose CT aligned in 86% of the cases (110/128). Chest X-ray aligned with standard-dose CT in 73% of the cases (92/126).

## Results

### Study population

**Fig. 1:**
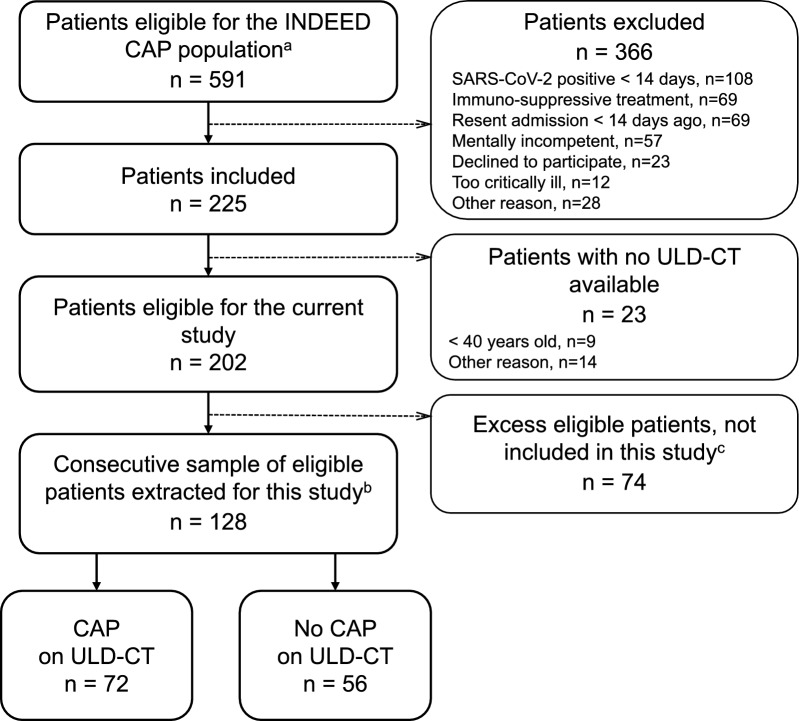
Flow of patients ^a^From the inclusion site at Hospital Lillebaelt, Kolding ^b^Patients included 8th April 2021—9^th^December 2021 ^c^Patients included between 1st March 2021—31th March 2021 & 10th December—25th February 2022 (March avoided to match availability of project blood samples.) INDEED: “Infectious Diseases in Emergency Departments” project CAP: Community-acquired pneumonia ULD-CT: Ultralow-dose computed tomography

The required sample of 128 patients was extracted from the INDEED population [16] (Fig. 1). Baseline characteristics of the patients are presented in Table 2. All ULD-CTs were performed within six hours of the patients’ arrival at the hospital (median 2.4 h). In 56% of the cases, CAP was radiologically present. Seventy-three percent of the ULD-CTs were deemed of sufficient quality to visualise any potential pathology. Only one ULD-CT was considered insufficient for diagnostics. Body mass index above 40 (4% of the patients) was always associated with reduced image quality.

**Table 2:** Characteristics of the study population

Demographics	Total n = 128
Age (years), median(IQR)	74 (63–82)
Male, n(%)	66 (52%)
Body Mass Index, median(IQR)^a^	26 (24–30)
Chronic pulmonary disease, n(%)^b^	53 (41%)
*Symptoms & finding*	
Dyspnoea, n(%)	91 (71%)
Cough, n(%)	98 (77%)
Expectoration, n(%)	80 (63%)
History of fever/fever symptoms, n(%)	87 (68%)
Temperature ≥ 38 or < 36 degrees Celsius in the ED, n(%)	37 (29%)
*Triage level*^*c*^*, n(%)*^*d*^	
Resuscitation	11 (9%)
Emergent	40 (33%)
Urgent	61 (50%)
Non-urgent	11 (9%)
*ULD-CT*	
Presence of CAP on ULD-CT by radiologist’s assessment, n(%)	72 (56%)
Hours from admission to hospital until the scan was performed, median(IQR)^d^	2.4 (1.8–3.1)
*ULD-CT image quality, assessed by radiologist, n(%)*	
Poor/non-diagnostic. Not possible to diagnose or exclude any pathology	1 (1%)
Suboptimal. Possible to diagnose or exclude rough pathology	33 (26%)
Optimal. Any potential pathology can be visualized, but resolution is imperfect	94 (73%)
Near perfect	0 (0%)

Percentages were rounded to the nearest whole number, so the total may not equal exactly 100%. ULD-CT: Ultralow-dose computed tomography.^a^ Missing = 33 (26%)—Data not available from the records or from interview.^b^ Including (but not limited to) chronic obstructive pulmonary disease, asthma, current or prior lung cancer, pulmonary fibrosis.^c^ Danish Emergency Process Triage [21] based on vital signs at arrival. ^d^ Missing values = 5

### Diagnostic accuracy

All clinicians assessed all 128 cases over a six-week period, starting immediately after the training course.

Seventy-eight percent of the clinicians’ CAP assessments corresponded to the radiologist’s assessment. Cross-tabulations are available in Additional file 3.

The diagnostic accuracy parameters are presented in Table 3. The clinicians’ overall sensitivity was 83% (95%CI: 77–88), specificity was 70% (95%CI: 59–81). Positive predictive value was 80% (95%CI: 74–84), negative predictive value was 78% (95%CI: 73–82). No statistically significant difference in diagnostic accuracy related to the clinicians’ level of experience was observed. From the point estimates, junior clinicians’ accuracy tended to be slightly better compared to consultants. The clinicians’ individual sensitivity ranged from 62 to 94%, specificity ranged from 30 to 89%.

**Table 3:** The clinicians’ diagnostic accuracy and interrater reliability in identifying community-acquired pneumonia on ultralow-dose CT

	Diagnostic accuracy parameters				Inter-rater reliability	
	Sensitivity % (95% CI)	Specificity % (95% CI)	PPV % (95% CI)	NPV % (95% CI)	Kappa^a^ (95% CI)	Percentage agreement % (95% CI)
All clinicians (n = 10)	83 (77–88)	70 (59–81)	80 (74–84)	78 (73–82)	0.54 (0.46–0.61)	78 (74–81)
Junior clinicians^b^ (n = 5)	86 (79–91)	72 (60–81)	80 (74—85)	80 (75—84)	0.61 (0.52—0.69)	82 (77–86)
Consultants^c^ (n = 5)	81 (68–89)	70 (47–86)	79 (68–87)	76 (68–83)	0.45 (0.36–0.54)	74 (69–78)

*CI: Confidence interval. PPV: Positive predictive value. NPV: Negative predictive value*

^*a*^*Conger’s kappa, a generalization for multiple raters *[18]

^*b*^Less than one year post graduate

^*c*^*Within internal and/or emergency medicine*

Sub-analyses using only cases with a better image quality, patients without a chronic pulmonary disease diagnosis, or assessments with high confidence (5–7 on the Likert scale) all resulted in slightly increased specificity. Maximum specificity was 77% for junior clinicians on cases without chronic pulmonary disease. Sensitivity stayed unchanged for consultants. Junior clinicians’ sensitivity increased to 89% when they were confident in their assessments (sub-analysis estimates are available in Additional file 4).

### Rater reliability

Inter-rater reliability among all clinicians was “moderate” (Table 3), kappa = 0.54 (95%CI: 0.46–0.61), and percent agreement = 78% (95%CI: 74–81). Kappa estimates and percent agreement were better among junior clinicians and had more narrow confidence intervals.

Intra-rater reliability was “almost perfect” for nine clinicians and “moderate” for one clinician. The average intra-rater kappa value was 0.87, average intra-rater percent agreement was 93.5%.

### Clinicians’ confidence and time consumption

**Fig. 2:**
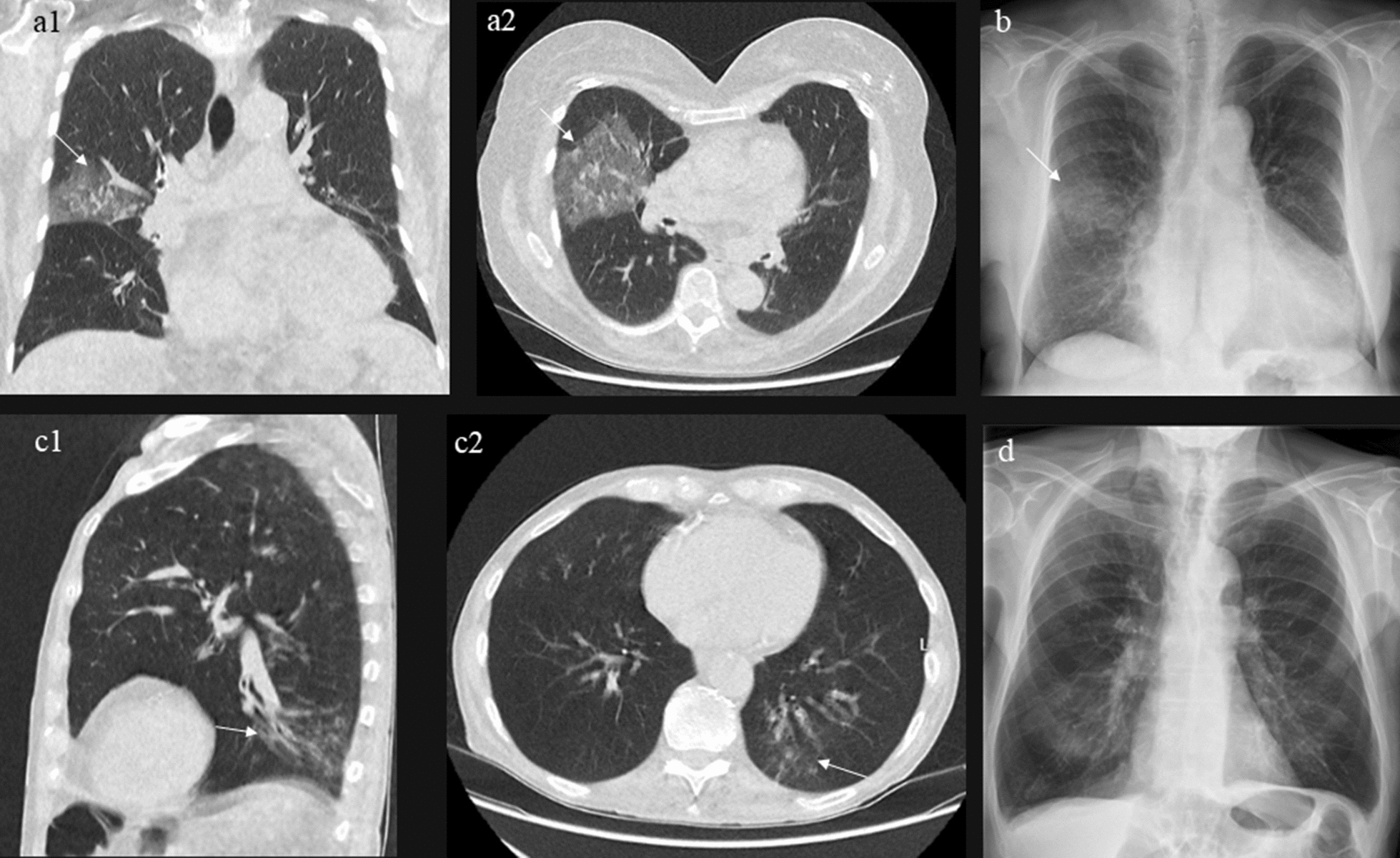
Examples of images from the study population (**a**) Ultralow-dose computed tomography (ULD-CT) of the chest from a patient with an opacity consistent with community-acquired pneumonia (CAP) in the right lobe, which is also seen in the corresponding chest radiograph (**b**). (**c**) ULD-CT of the chest from a patient with a left lower lobe opacity consistent with CAP. This opacity was not initially identified on the corresponding chest radiograph by the reporting radiologist (**d**). After reviewing the ULD-CT and comparing it with the chest radiograph, a small pneumonia could be suspected upon a second examination of the images (**c**, **d**)

On the 7-point Likert scale, median confidence was 6 (IQR: 5–7) in assessments regarding CAP presence for both clinician groups. Median time consumption for each assessment, including registration, was 2 min (IQR 1–3) for consultants and 2 min (IQR: 2–3) for juniors (Fig. 2).

## Discussion

In this study, five consultants, five junior clinicians, and one radiologist assessed 128 unique ULD-CTs for CAP changes. Clinicians’ assessments showed good consensus (78%) with the radiologist, and clinicians’ confidence in their assessments was good. The clinicians’ overall sensitivity was 83% (95%CI: 77–88), specificity was 70% (95%CI: 59–81), and positive and negative predictive values were high. No statistically significant difference in diagnostic accuracy was found between juniors and consultants. Interrater reliability between clinicians was only moderate, with a non-significant tendency towards better reliability among junior clinicians compared to consultants.

To our knowledge, this is the first study investigating the diagnostic accuracy of clinicians’ assessments of ULD-CT targeted primarily at a CAP diagnosis. One study previously examined clinicians’ agreement in assessing ULD-CTs from ED patients presenting with dyspnoea [22]. The prevalence of pneumonia in their group was low (11%), and no comparable diagnostic accuracy estimates were available. Similar to this study, they reported a tendency towards better interrater reliability among junior clinicians (kappa = 0.66) compared to consultants (kappa = 0.33). A possible explanation of this trend is that the junior doctors form a more uniform group.

Previous studies reported that radiologist-reported reduced-dose CT improved the clinicians’ diagnostic certainty in patients admitted with suspected pneumonia, especially in terms of ruling out pneumonia in patients with an intermediate probability of having pneumonia prior to CT [7, 15]. In the current study, clinicians’ specificity was only moderate, and 13% of the assessments were false-positive. This suggests difficulties for clinicians to independently rule out pneumonia from pneumonia-negative ULD-CTs, thereby limiting the degree to which unnecessary antibiotic treatments can be reduced. Yet, it should be emphasised that imaging is not a stand-alone diagnostic tool in CAP [23]. Thus, this study primarily represents an isolated view of diagnosis with an unreported ULD-CT compared to having a radiological report.

We identified no recent, comparable studies evaluating clinicians’ diagnostic accuracy in assessing chest radiographs for pneumonia. A study from 1994 found that 66–72% of clinicians’ assessments for pneumonia on 15 chest radiographs were in agreement with the reference assessments by radiologists [24], which is slightly lower compared to our findings from ULD-CT. Previous studies on clinicians’ general assessments of chest radiographs indicated deficient skills, especially among junior doctors [25, 26]. In the current study, there was no effect of clinical experience on diagnostic accuracy. From this, it could be hypothesised that this study’s results represent a baseline level of clinicians’ accuracy, with room for improvement over time if the investigation becomes more widely used and clinicians’ gain more experience in assessing ULD-CT’s.

A strength of this study was the training and testing of the clinicians before ULD-CT assessments. This ensured equal basic qualifications. The course was pragmatic, interactive, and of reasonable duration, thus representing a realistic offer to medical staff outside a study setting. The training course did not cover all types of pneumonic changes in detail. Therefore, some disagreement was expected due to the radiologist’s ability to more confidently identify a broader spectrum of pneumonic changes. Possibilities exist to expand training and learning, for instance, by increasing the duration and intensity of the training programme or with ongoing feedback, which could be achieved with a possibility to follow up on the radiologists’ reports.

This study presents relevant data to support considerations of implementing chest ULD-CT for in-hospital infection diagnosis. Yet, several issues still need further clarification, including better clarification of the impact on radiological capacity, the amount and relevance of additional incidental findings, and the effect on patient management.

No clinical data were available for the clinicians and the radiologist while interpreting ULD-CTs. This was a strength in terms of enhancing objectivity of image interpretation. However, patient history and diagnostic expectations affect diagnostic performance among radiologists [27], and clinicians’ assessments will probably be influenced by other clinical data in a real setting as well. Thus, the same degree of objectivity might not apply outside the study setting. Further, no access to previous patient imaging can be viewed as a limitation, as previous images could assist in correctly identifying acute changes, thereby increasing diagnostic accuracy for acute CAP-related findings.

Another limitation was the exclusion criteria set for the INDEED population. More than half of the patients suspected of having CAP were excluded. The main reason was the SARS-CoV-2 infection, which seems reasonable in a post-pandemic context. Yet, especially the exclusion of patients with immunosuppressive treatments or recent admissions, could affect generalisability as ULD-CTs from these patients could be more difficult to assess.

Occurrence of some degree of inter-radiologist variability in image readings is well known, including from ULD-CT readings [22] and pneumonia diagnoses from chest radiographs [28, 29]. Thus, some classification bias could be present in this study, especially because early-stage CAP cases are represented in the study population. Further, a chest ULD-CT reported by one ED radiologist cannot be regarded as a gold standard for diagnostic imaging in CAP. It represents a pragmatic reference standard comparable to what is available in the ED.

Patients’ BMI impacts the possibilities for radiation dose reduction [30]. This study revealed that a BMI > 40 was always associated with reduced image quality. This indicates that for very obese patients, ULD-CT is not the best investigation, and adjustments in radiation doses or scanning protocols may be necessary.

## Conclusions

This study found that clinicians could assess chest ULD-CTs for CAP with high, but not perfect, diagnostic accuracy using an ED radiologist’s assessments as reference standard. Interrater reliability among clinicians was moderate. A higher level of clinical experience was not associated with better accuracy or interrater reliability.

### Supplementary Information

Additional file 1: Index test assessment template.

Additional file 2: Reference standard assessment template; Parts of the radiological INDEED project ULD-CT assessment template used for this study.

Additional file 3: Cross-tabulations of test result proportions.

Additional file 4: Diagnostic accuracy; subgroup analyses.

## Data Availability

The dataset used in the current study is available from the corresponding author on reasonable request.

## Abbreviations

CT: Computed tomography
ULD-CT: Ultralow-dose computed tomography
CAP: Community-acquired pneumonia
ED: Emergency department
mSv: Millisievert
PACS: Picture archiving and communication system
REDCap: Research Electronic Data Capture
GEE: Generalised estimating equations
PPV: Positive predictive value
NPV: Negative predictive value
CI: Confidence interval
