# Supplementary material for: Can clinicians identify community-acquired pneumonia on ultralow-dose CT? A diagnostic accuracy study
Source: Scand J Trauma Resusc Emerg Med. 2024 Aug 7;32:67. doi: 10.1186/s13049-024-01242-w (PMC11304923; doi:10.1186/s13049-024-01242-w)
Supplement: Supplementary file 1 — Additional file 1: Index test assessment template. [file 13049_2024_1242_MOESM1_ESM.docx]

#### Additional file 1: Index test assessment template

Outline of the assessment completed by the clinicians for each ULD-CT case on a web-based registration template:

| Start time for assessment | Set by a click |
| --- | --- |
| ULD-CT diagnosis | - Normal - Pneumonic opacity - Pulmonary oedema - Pleural effusion - Pneumothorax - Other |
| How sure are you on a score from 1-7 on your assessment regarding pneumonic opacity? (1=very unsure, 7=absolutely sure) | - 1 - 2 - 3 - 4 - 5 - 6 - 7 |
| *Additional question if checked in “pneumonic opacity”:*  Placement of pneumonic opacity? | - Left - Right - Bilateral |
| *Additional question if checked in “pleural effusion”:*  Bilateral effusion? | - Yes - No |
| *Additional question if checked in “pneumothorax”:*  Pneumothorax side? | - Left - Right - Bilateral |
| *Additional text field for optional elaboration if checked in “other”* | (optional text) |
| End time for assessment | Set by a click |
