# Supplementary material for: Can clinicians identify community-acquired pneumonia on ultralow-dose CT? A diagnostic accuracy study
Source: Scand J Trauma Resusc Emerg Med. 2024 Aug 7;32:67. doi: 10.1186/s13049-024-01242-w (PMC11304923; doi:10.1186/s13049-024-01242-w)
Supplement: Supplementary file 2 — Additional file 2: Reference standard assessment template; Parts of the radiological INDEED project ULD-CT assessment template used for this study. [file 13049_2024_1242_MOESM2_ESM.docx]

#### Additional file 2: Reference standard assessment template

Parts of the radiological INDEED project ULD-CT assessment template used for this study.

| ULD-CT image quality | - 1. Poor image quality: it is not possible to recognise any anatomical structures - 2. Some anatomical structures can be visualised, but it is still not possible to diagnose or exclude any pathology - 3. Suboptimal image quality: Some anatomical structures can be visualised, and it is possible to diagnose or exclude rough pathology - 4. All relevant anatomical structures and any potential pathology can be visualised, but still the resolution of the image are not perfect. - 5. All relevant anatomical structures and any potential pathology can be visualised and the resolution of the picture are near perfect |
| --- | --- |
| Suspected diagnoses based on ULD-CT  (several diagnoses may be chosen) | - Normal findings - Pneumonia - Non-cardiogenic pulmonary oedema - Cardiogenic pulmonary oedema - Pneumonia with parapneumonic effusion - Empyema - Pleural effusion of unknown origin - Pulmonary embolism - Pneumothorax - COPD (Chronic obstructive pulmonary disease - Asthma - Interstitial lung disease - Malignancy - Other |
| *Additional text field for optional elaboration if checked in “other”* | (text) |

The reference standard for the primary outcome was positive if the radiologist had checked either “Pneumonia” or “Pneumonia with parapneumonic effusion” or “other”, and the description was suggestive of pneumonia under “suspected diagnoses based on ULD-CT”. There were no checks in Empyema in this study population.
