# Supplementary material for: Can clinicians identify community-acquired pneumonia on ultralow-dose CT? A diagnostic accuracy study
Source: Scand J Trauma Resusc Emerg Med. 2024 Aug 7;32:67. doi: 10.1186/s13049-024-01242-w (PMC11304923; doi:10.1186/s13049-024-01242-w)
Supplement: Supplementary file 3 — Additional file 3: Cross-tabulations of test result proportions. [file 13049_2024_1242_MOESM3_ESM.docx]

#### Additional file 3: Cross tabulation of test result proportions

All clinicians

| Pneumonia,  reference standard  (128 assessments) | Pneumonia, index test, all clinicians  (128x10 assessments) | |  |
| --- | --- | --- | --- |
|  | **No** | **Yes** | **Total** |
| No | 31.0 % | 12.7 % | 43.7 % |
| Yes | 9.4 % | 46.9 % | 56.3 % |
| Total | 40.4 % | 59.6 % | 100 % |

By level of clinical experience

| Pneumonia,  reference standard  (128 assessments) | Pneumonia, index test, junior clinicians  (128x5 assessments) | |  |
| --- | --- | --- | --- |
|  | **No** | **Yes** | **Total** |
| No | 31.4 % | 12.3 % | 43.7 % |
| Yes | 8.0 % | 48.3 % | 56.3 % |
| Total | 39.4 % | 60.6 % | 100 % |

| Pneumonia,  reference standard  (128 assessments) | Pneumonia, index test, consultants  (128x5 assessments) | |  |
| --- | --- | --- | --- |
|  | **No** | **Yes** | **Total** |
| No | 30.6 % | 13.1 % | 43.7 % |
| Yes | 10.8 % | 45.5 % | 56.3 % |
| Total | 41.4 % | 58.6 % | 100 % |
