# Supplementary material for: Can clinicians identify community-acquired pneumonia on ultralow-dose CT? A diagnostic accuracy study
Source: Scand J Trauma Resusc Emerg Med. 2024 Aug 7;32:67. doi: 10.1186/s13049-024-01242-w (PMC11304923; doi:10.1186/s13049-024-01242-w)
Supplement: Supplementary file 4 — Additional file 4: Diagnostic accuracy; subgroup analyses. [file 13049_2024_1242_MOESM4_ESM.docx]

#### Additional file 4: Diagnostic accuracy; subgroup analyses

| Subgroup |  | Junior clinicians | Consultants |
| --- | --- | --- | --- |
| Good image quality  (n=94 cases = 470 assessments pr. group) | Sensitivity, %(95%CI) | 87 (83-91) | 81 (70-88) |
|  | Specificity, %(95%CI) | 76 (67-83) | 75 (52-89) |
| No chronic pulmonary disease  (n= 75 cases = 375 assessments pr. group) | Sensitivity, %(95%CI) | 84 (78-88) | 80 (65-89) |
|  | Specificity, %(95%CI) | 77 (61-88) | 74 (74-90) |
| High confidence, 5-7 on the Likert scale  (juniors: n=543 assessments)  (consultants: n=574 assessments) | Sensitivity, %(95%CI) | 89 (83-93) | 81 (67-90) |
|  | Specificity, %(95%CI) | 75 (65-83) | 73 (50-88) |
